# Making the Most of Lateral Flow Immunochromatographic Tests: An Efficient Protocol to Recover DNA

**DOI:** 10.3390/mps7010008

**Published:** 2024-01-15

**Authors:** Sara C. Zapico, Gabriela Roca

**Affiliations:** 1 Department of Chemistry and Environmental Science, New Jersey Institute of Technology, Newark, NJ 07102, USA; 2 Anthropology Department and Laboratories of Analytical Biology, National Museum of Natural History, Smithsonian Institution, Washington, DC 20560, USA; 3 SERATEC^®^ Gesellschaft für Biotechnologie mbH, 37079 Göttingen, Germany; gabriela.roca@seratec.com

**Keywords:** lateral flow immunochromatographic (LFI) tests, body fluids, silica-based DNA extraction, fluorescent quantification, human-specific quantification, STR profile

## Abstract

Lateral flow immunochromatographic (LFI) tests are widely used in both biomedical and forensic sciences for different applications. In forensic sciences, their main use is to detect body fluids at crime scenes. However, there are situations in which the amount of potential biological evidence is so low that DNA extraction is favored with respect to the identification of body fluids. Here, an efficient and quick protocol is presented to integrate the detection of body fluids through LFI with DNA extraction from a sample swab and buffer, providing a complete characterization of the biological evidence. This protocol is a modification of a general DNA extraction silica-based kit, whose main application is for blood and tissues. Thus, it could be carried out in different settings (forensic labs, hospitals, other testing labs) without the necessity of buying a specific kit for swabs. The validation of this protocol is supported by the results presented here and previous publications from our group, obtaining DNA in good quantity and with good quality. This proves the potential application of the protocol in both forensic scenarios, to fully characterize biological evidence, and biomedical settings, to molecularly confirm the results of LFI tests.

## 1. Introduction

The detection of body fluids at crime scenes is the first step to potentially discover biological materials. After confirming the presence of the body fluid, the next step is to individualize this evidence, identifying the person who left the body fluid behind by DNA extraction, quantification, and the creation of an STR profile, leading to finding a match with a possible suspect.

Lateral flow immunochromatographic (LFI) tests have been widely used in biomedical and forensic sciences for the speed of obtaining results, which is the fastest available. These tests are based on antigen–antibody reactions. The tests use a mobile and stationary monoclonal antibody against the protein of interest, which will form a visible pink line if the protein is present [1]. In biomedical sciences, the most recent application has been to detect COVID-19 [2,3,4], though they are also used for the diagnosis of other microbiological diseases [5,6,7]. In forensic sciences, these tests are designed to detect body fluids at a crime scene or in a lab, blood, semen, saliva, menstrual blood, and urine [1,8,9,10]. 

In contrast to biomedical science samples, biological samples from crime scenes are in low quantity, and sometimes, the forensic scientist has to decide between performing these LFI tests and losing part of the sample or directly sending the sample for DNA analysis. If the latter is chosen, there are some disadvantages: at the monetary level, it is possible that the sample is not a biological sample, and DNA extraction and quantification reagents are wasted in the process. Some information will be missing; e.g., semen and saliva look almost the same, and if they are not identified by the tests and only the STR profile is obtained, this could hamper the reconstruction of the crime [11,12]. As a result, the identification of the body fluid is as important as DNA profiling. Additionally, DNA extraction is a destructive process; when it is performed, it is not possible to come back to identify the proteins. 

There have been some attempts to combine these two objectives and be able to identify the body fluid and extract a DNA profile from the sample [12,13,14,15] and from the LFI test strips [16], obtaining most of the sample without losing it. Our previous publications also demonstrated successful DNA recovery and profiling from a sample swab [17,18] and LFI test strips [19]. These protocols could be applied to biomedical sciences to confirm the diagnosis of LFI tests [5,7,20]. 

Thus, the present protocol describes an efficient method to extract DNA from a sample swab while also assessing the DNA yield and profiling from these samples. This protocol is supported by our previous publications [17,18,19]. As a result, with the publication of this detailed protocol, we are hoping it could be useful for both the forensic and biomedical science communities based on the widespread application of LFI tests. 

## 2. Experimental Design

A volume of 50 µL of fresh human saliva samples (three replicates per fabric and time) were deposited on three types of fabrics (denim, cotton, and polyester) and left at room temperature in a biological safety cabinet. After 24 h, the samples were recovered with one cotton swab (sterile, wood-shaft, cotton-tipped applicators from McKesson, Richmond, Virginia) moisturized with SERATEC^®^ extraction buffer (SERATEC^®^, Göttingen, Germany) and applied to the sample by 3–5 circular movements. As a positive control, a buccal swab was used. Two negative controls were included, one using a cotton swab moisturized with SERATEC^®^ extraction buffer (SERATEC^®^, Göttingen, Germany), and applied to an area of the fabric without saliva by 3–5 circular movements. The other negative control was the swab only. Then, swabs were incubated in agitation at room temperature in 300 µL of extraction buffer from the SERATEC^®^ Amylase test (SERATEC^®^, Göttingen, Germany) for 10 min. After that, three drops (120 µL) of the buffer were added to the SERATEC^®^ Amylase LFI test cassettes. After 10 min, the results were recorded. A valid positive result included two pink bands, the control band (always present) and the test band. The tube containing the swab and the remaining extraction buffer was stored at −20 °C until DNA extraction was performed. Positive LFI test cassettes were stored at room temperature until the DNA extraction procedure. 

### 2.1. Materials

- Fresh human saliva samples directly collected by spitting in an Eppendorf tube. Based on our current experiments, other body fluids like semen and blood can be used.
- Sterile, wood-shaft, cotton-tipped applicators from McKesson (Richmond, Virginia).
- SERATEC^®^ Amylase test (SERATEC^®^, Göttingen, Germany).
- SERATEC^®^ extraction buffer (SERATEC^®^, Göttingen, Germany). Components: 8.0 g of NaCl; 0.2 g of KCl; 1.44 g of Na_2_HPO_4_·2H_2_O; 0.24 g of KH_2_PO_4_; 0.1 mL of 10 wt% NaN_3_; pH 7.4 in 1 L of distilled water.
- DNeasy Blood & Tissue kit (Qiagen^®^, Hilden, Germany).
- Molecular biology-grade ethanol, 99% (Sigma-Milipore, Saint Louis, MO, USA).
- Qubit dsDNA HS (high-sensitivity) Assay Kit (Life Technologies, Carlsbad, CA, USA).
- Thin-wall, clear, 0.5 mL PCR tubes (Genessee Scientific, Morrisville, NC, USA).
- 96-well plates (Thermo Fisher Scientific, Waltham, MA, USA).
- PowerQuant System (Promega Corporation, Madison, WI, USA).
- Promega PowerPlex Fusion 6C System (Promega Corporation, Madison, WI, USA).
- Hi-Di formamide (Thermo Fisher Scientific, Waltham, MA, USA).

### 2.2. Equipment

- Biological safety cabinet (Thermo Fisher Scientific, Waltham, MA, USA).
- Vortex (Thermo Fisher Scientific, Waltham, MA, USA).
- Thermal mixer with blocks (Thermo Fisher Scientific, Waltham, MA, USA).
- Centrifuge (Thermo Fisher Scientific, Waltham, MA, USA).
- Qubit Fluorometer 3.0 (Thermo Fisher Scientific, Waltham, MA, USA).
- QuantStudio 5 (Thermo Fisher Scientific, Waltham, MA, USA).
- ProFlex PCR system (Thermo Fisher Scientific, Waltham, MA, USA).
- SeqStudio (Thermo Fisher Scientific, Waltham, MA, USA).

### 2.3. Software

- Promega PowerQuant Analysis Software Version: 4.8.0.0 (Promega Corporation, Madison, WI, USA).
- Microsatellite Analysis Software on Thermo Fisher Cloud (Thermo Fisher Scientific, Waltham, MA, USA).

## 3. Procedure

### 3.1. DNA Extraction from the Swab and Extraction Buffer (Figure 1)

1. Keep the swab on the Eppendorf tube. Complete to 400 µL with SERATEC^®^ extraction buffer.
2. From the DNeasy Blood & Tissue kit, add to the buffer 20 µL of Proteinase K and 400 µL of Reagent AL. Vortex for 15 s.
3. Incubate and shake at 56 °C for 10 min on the thermal mixer with blocks.
4. Add 400 µL of Ethanol. Vortex for 15 s.
5. Transfer 700 µL of the mixture to the DNeasy Blood and Tissue kit column. Centrifuge at 6000× *g* (8000 rpm) for 1 min. Discard the flow-through liquid. Repeat this step with the rest of the mixture. The DNA is now attached to the column.
6. Add to the column 500 µL of Buffer AW1. Centrifuge at 6000× *g* (8000 rpm) for 1 min. Discard the flow-through liquid.
7. Add to the column 500 µL of Buffer AW2. Centrifuge at 20,000× *g* (14,000 rpm) for 3 min. Discard the flow-through liquid.
8. In order to evaporate all ethanol, centrifuge the column at 20,000× *g* (14,000 rpm) for 1 min.
9. After cleaning the DNA, perform the elution/release of DNA from the column by adding to the column 50 µL of buffer AE (or nuclease-free water). Incubate for 1 min at RT. Then, centrifuge at 6000× *g* (8000 rpm) for 1 min.
10. The DNA is released.

**Figure 1:**
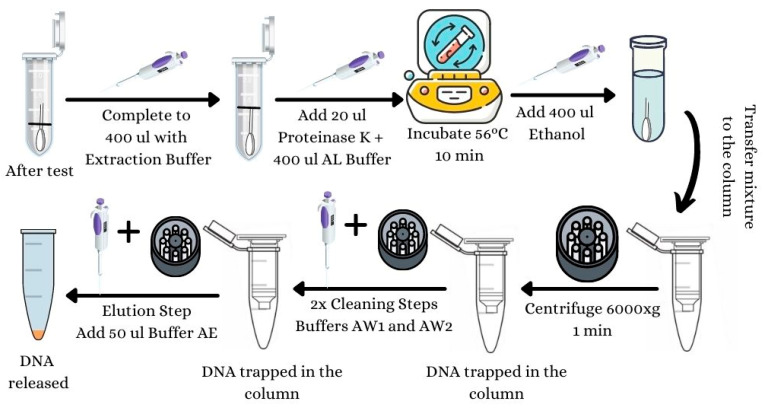
DNA extraction procedure.

### 3.2. Quantification of Total DNA with Qubit dsDNA HS

1. Bring all the kit components and samples to room temperature (RT).
2. Prepare a Working Solution (WS) composed of Qubit^®^ dsDNA HS Buffer and Qubit^®^ dsDNA HS Reagent (concentrated 200×), considering that each sample will be diluted in approximately 200 µL of this solution.
3. The kit includes two DNA standards, Standard #1, 0 ng/µL DNA in TE buffer, and Standard #2, 10 ng/µL DNA in TE buffer.
4. Mix 190 µL of the WS with 10 µL of each standard in thin-wall, clear, 0.5-well tubes.
5. For the samples, although the manufacturer’s protocol indicates that it is possible to use as little as 1 µL of the sample (between 1 and 10 µL), from our experience, 2 µL works better. Thus, mix 2 µL of each sample with 198 µL of the WS in thin-wall, clear, 0.5-well tubes.
6. Mix all the tubes by vortexing.
7. Incubate at room temperature for 2 min.
8. Read the sample on the Qubit Fluorometer 3.0, selecting dsDNA HS. Clean the tubes before introducing them on the instrument.
9. After the reading, indicate how much sample you used to obtain the final concentration of your sample.

### 3.3. Specific Human Quantification with the PowerQuant System

1. The day before the quantification, defrost the standard (PowerQuant male gDNA standard) and the dilution buffer and keep them at 4 °C.
2. Prepare serial dilutions of the PowerQuant male gDNA standard with the PowerQuant dilution buffer, starting with the original 50 ng/µL, 2 ng/µL, 0.08 ng/µL, and 0.0032 ng/µL (Table 1).

3. Prepare the Master Mix considering the number of samples and the standards. Both samples and standards should be tested per duplicate. The Master Mix includes the PowerQuant Master Mix (concentrated 2×), the PowerQuant Primer/Probe/IPC Mix (concentrated 20×), and water amplification grade, totaling a volume of 18 µL, as depicted in Table 2.

4. Vortex the Master Mix and distribute 18 µL on a 96-well plate.
5. Add 2 µL of the standards/samples to the plate.
6. Seal the plate and centrifuge briefly.
7. Load the plate onto the QuantStudio.
8. Introduce the appropriate parameters to the QuantStudio: block type, 96-well 0.2 mL block; experiment type, standard curve; chemistry, TaqMan Reagents; run mode, standard.
9. In Experiment Method, enter 20 uL for “Volume” and set up the parameters for the thermocycler: Hold Stage, 98 °C 2 min; PCR stage: Step 1: 98 °C 15 s. and Step 2: 62 °C 35 s for 39 cycles. The ramp rate for all three steps is 2.44 °C/s.
10. In “Create a Run Template”, assign the following targets to all samples: Autosomal, with a reporter PQ_FAM and quencher NFQ-MGB; Y, with a reporter PQ_CFG540 and quencher NFQ-MGB; Degradation, with a reporter PQ_Q670 and quencher NFQ-MGB; and IPC, with a reporter PQ_TMR and quencher NFQ-MGB. The Task for autosomal, Y, and degradation targets should be “S”, and for IPC, it should be “U”.
11. In the Samples section, add the standards’ and the samples’ names and select the appropriate wells.
12. In the Quick Setup tab, in Plate Attributes, select PQ_CXR as the passive reference.
13. In the CT Settings tab, select each target and uncheck the Default Setting box and the Automatic Threshold box. Enter the following values for the Threshold: Autosomal, 0.2; Degradation, 0.2; IPC, 0.03; Y, 0.2. Select Apply.
14. When all the parameters are set up, click on Next, and then Start Run.
15. Data analyses will be carried out with the PowerQuant Analysis Software.
16. For the standard curve, the minimum acceptable value for R-Squared for the three targets is 0.99; for the Slope, it is −3.6, and the maximum acceptable value is −3.1. Including the Y-intercept is optional.
17. Establishing a Threshold value and Sample Assessment Message should be customized based on internal validation studies conducted by the lab. The Inhibitor threshold indicates the minimum IPC shift value at which you may expect to encounter inhibition. The Male/Female threshold specifies the minimum Autosomal/Y ratio indicative of potential male/female mixture. The Degradation threshold specifies the minimum autosomal/degradation ratio indicative of a potentially degraded DNA sample.
18. Once the csv file from QuantStudio is loaded onto the PowerQuant Software, the data will be analyzed, according to the set-up parameters. The Quantity Map will give the concentration of autosomal, Y, and IPC in ng/µL. The Ratio Map will give the Sample Assessment parameters’ values. For proceeding to the next step of STR amplification, the concentration of autosomal DNA in ng/µL is chosen.

### 3.4. Amplification of STRs Using the PowerPlex Fusion 6C System

1. Centrifuge pre-amplification component tubes briefly, and vortex for 15 s before each use.
2. From the samples, between 0.5 and 1 ng DNA can be used. A positive control is included, the 2800M Control DNA, from this 1 ng of DNA should be amplified.
3. Prepare the Master Mix considering the number of samples, positive and negative controls (with Water, Amplification Grade). Add 1 or 2 reactions to this number. This Master Mix consists of 5 µL of PowerPlex Fusion 6C 5× Master Mix, 5 µL PowerPlex Fusion 6C 5× Primer Pair Mix, and Water, Amplification Grade to a final volume of 25 uL. Vortex this mix for 5–10 s and then add to each tube.
4. Add the template DNA (up to 15 µL) considering that the final volume is 25 µL.
5. Briefly centrifuge the tubes.
6. Set up the tubes in the Proflex and program the following parameters: 1 cycle of 96 °C 1 min; 29 cycles of 96 °C 5 s. and 60 °C 1 min; 1 cycle 60 °C 10 min; Hold 4 °C.
7. After the reaction, the samples can be stored at −20 °C or directly proceed with fragment analysis.

### 3.5. Fragment Analysis on the SeqStudio

1. Centrifuge post-amplification component tubes briefly, and vortex for 15 s before each use.
2. Apart from the previous samples, two allelic ladders are included in the fragment analysis to be able to interpret the bins.
3. Prepare a loading cocktail considering the number of samples, positive and negative controls, and two allelic ladders. Add 1 or 2 reactions to this number. This cocktail consists of 0.5 µL of WEN ILS 500 and 9.5 µL of Hi-Di formamide. Vortex the cocktail and pipet 10 µL in each tube.
4. Add 1 µL of amplified sample or allelic ladder in the tubes. Centrifuge briefly.
5. Denature samples at 95 °C for 3 min in the thermoblock, and chill on a freezer block for 3 min.
6. Transfer the samples to the 96-well plate and cover the wells with appropriate septa.
7. Introduce the plate into the SeqStudio.
8. Create a new plate in the instrument, selecting Fragment Analysis as the application.
9. Select the appropriate wells and include as a Run Module “FragAnalysis”; Size Standard “Promega ILS 500”; and Dye Set “Promega 6C”. Include the sample name and indicate if they are a sample, positive control, negative control, or allelic ladder.
10. The running parameters are the following: 7 s injection time; 1200 volts injection voltage; 1440 s run time; 9000 volts run volt.
11. Analyze the data in the Microsatellite Analysis software on Thermo Fisher Cloud. Set up the parameters based on the available panels, bins, and size standards from Promega online.

## 4. Expected Results

### 4.1. Reagents

The reagents used were international standards, and the complete composition of them is normally disclosed in the manufacturer’s protocol. SERATEC^®^ Amylase tests were chosen due to their widespread use by police and law enforcement agencies worldwide. They have a minimum sensitivity of 50 mIU/mL, according the manufacturer’s website. Additionally, the formula for their extraction buffer is provided, making it easier to assess and apply the appropriate DNA extraction method, avoiding any interferences or inhibitions with the kit’s reagents. The DNEasy Blood & Tissue kit was chosen because of its simple and easy-to-use protocol, and previous publications from our lab demonstrated its successful performance with tough samples like teeth, food bite marks, and contact lenses, only requiring a modification of the protocol depending on the substrate [21,22,23].

### 4.2. Timeline

Depending on the number of samples, the DNA extraction protocol will take approximately between 1 and 2 h, or even less. This means that, on the same day, it would be possible to perform the LFI tests, DNA extraction, and quantification with both Qubit and PowerQuant. Then, the following day, PCR and fragment analysis could be completed. Thus, the whole protocol from LFI tests to STR profiles could take approximately between 2 and 3 days. 

### 4.3. DNA Concentration and Quality

**Figure 2:**
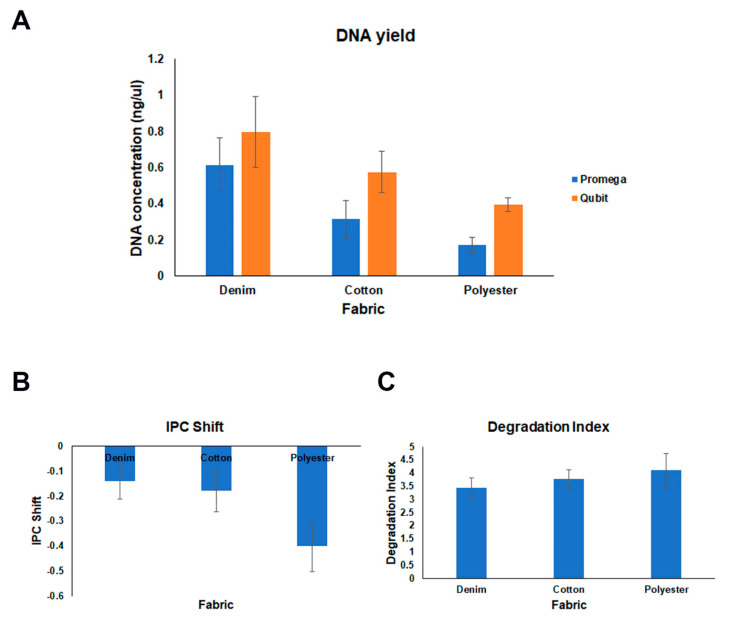
Assessment of DNA yield and quality. (**A**) DNA concentration based on Qubit and Promega PowerQuant quantifications. (**B**) IPC shift; ratio to assess the presence of PCR inhibitors. (**C**) Degradation Index; ratio to determine the level of degradation of DNA.

Figure 2A shows the concentrations obtained per fabric with both Qubit and PowerQuant. As expected, human-specific quantification was lower than fluorescent quantification. Despite this, human-specific quantification obtained per fabric was enough to perform the next step, the amplification of the STRs through PCR using 1 ng of the sample. Figure 2B depicts the results of the PowerQuant regarding the presence of inhibitors, with the parameter called IPC shift. As the graph shows, the values were negative; thus, no inhibitors were detected in the samples, even among the different types of fabrics. Figure 2C shows the assessment of DNA degradation, through the Degradation Index (DI). The values were between 3 and 4, indicating no degradation or minimum degradation of the DNA, matching with previous results from our lab. 

### 4.4. STR Profiles

**Figure 3:**
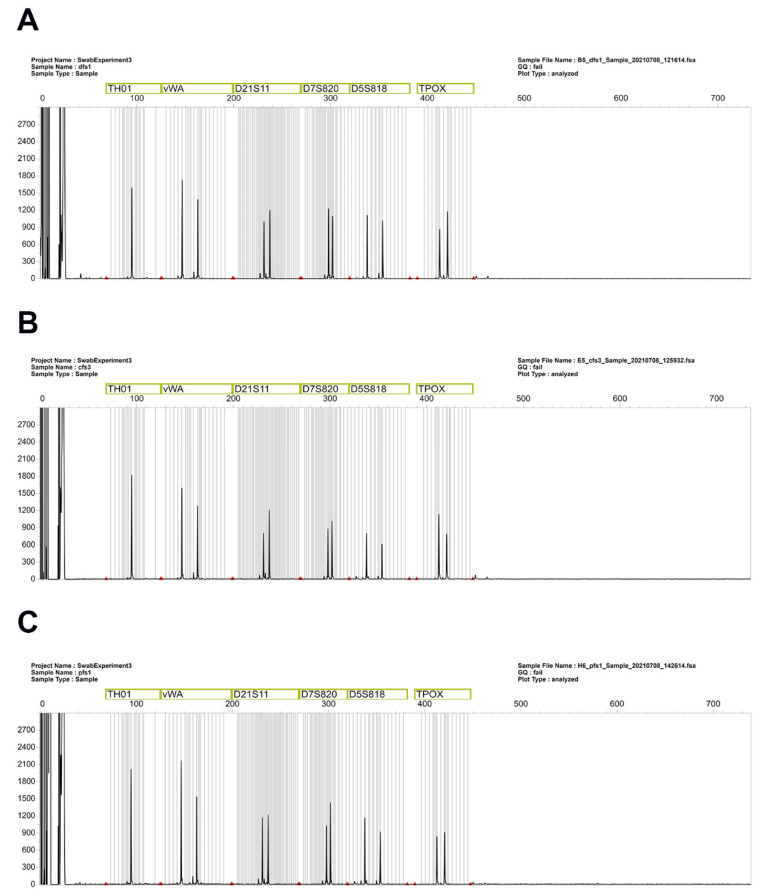
Examples of STR profiles obtained from samples deposited on (**A**) denim, (**B**) cotton, and (**C**) polyester.

The STR profiles obtained from all the samples were reproducible and of good quality. Figure 3 shows a comparison of one of the STR channels, demonstrating that, with slight variability, there were no differences in peak heights among the fabrics. Previous publications from our group assessed, as a measure of the quality of the DNA profiles, total peak height, peak height ratio, and interlocus balance, again demonstrating that, with variability among times, it is possible to obtain good-quality DNA profiles from saliva samples deposited on different fabrics [17,18]. 

## 5. Conclusions

A complete, quick, “step-by-step” protocol for DNA isolation from the sample swab and buffer remaining from LFI tests was presented. This protocol was carried out with a general silica-based kit, which was originally designed for DNA extraction from blood and tissues. Our protocol adapts the methodology of this kit to be able to use it with swabs, without the necessity to buy a specific kit for this kind of sample. As a result, it could be widely applied in hospitals and other labs, where their main samples are blood and tissues. Additionally, based on our current research, this protocol is also efficient for other body fluids like blood and semen. Although, this protocol has not been used yet to extract DNA from mixtures of semen and vaginal fluids, which could be encountered in sexual assault cases. This will require additional steps, e.g., using DTT for differential extraction, which could increase the time and the efficiency of the protocol. For clean, isolated body fluid samples, as shown by the results and our previous publications, this protocol demonstrated a 100% efficiency for DNA quantification and obtaining good-quality STR profiles. This is of utmost importance in forensic cases; the amount of sample could be so small that, sometimes, the investigator needs to decide between performing body fluid identification and directly sending the sample for DNA analysis. This protocol gives the chance to perform both while also avoiding the wasting of evidence that could be required later for cross-examination and/or case reviews. Finally, this protocol could be applied to biomedical sciences to confirm the diagnosis of diseases detected by LFI tests.

## Figures and Tables

**Table 1:** Serial dilutions of the standards.

Standard Concentration	Volume of Male gDNA Standard	Volume of PowerQuant Dilution Buffer
50 ng/µL	Undiluted standard	0 µL
2 ng/µL	4 µL of undiluted standard	96 µL
0.08 ng/µL	4 µL of 2 ng/µL dilution	96 µL
0.0032 ng/µL	4 µL of 0.08 ng/µL dilution	96 µL

**Table 2:** Components for one amplification reaction.

Reaction Component	Volume per Reaction
Water Amplification Grade	7 µL
PowerQuant 2× Master Mix	10 µL
PowerQuant 20× Primer/Probe/IPC Mix	1 µL
**Final Volume**	**18 µL**

## Data Availability

Data will be available upon request.
